# SparkRA: Enabling Big Data Scalability for the GATK RNA-seq Pipeline with Apache Spark

**DOI:** 10.3390/genes11010053

**Published:** 2020-01-03

**Authors:** Zaid Al-Ars, Saiyi Wang, Hamid Mushtaq

**Affiliations:** Computer Engineering Lab, Delft University of Technology, Mekelweg 5, 2628 CD Delft, The Netherlands; S.Wang-17@student.tudelft.nl (S.W.); H.Mushtaq@tudelft.nl (H.M.)

**Keywords:** GATK variant calling, RNA-seq, Apache Spark, scalability, computation time

## Abstract

The rapid proliferation of low-cost RNA-seq data has resulted in a growing interest in RNA analysis techniques for various applications, ranging from identifying genotype–phenotype relationships to validating discoveries of other analysis results. However, many practical applications in this field are limited by the available computational resources and associated long computing time needed to perform the analysis. GATK has a popular best practices pipeline specifically designed for variant calling RNA-seq analysis. Some tools in this pipeline are not optimized to scale the analysis to multiple processors or compute nodes efficiently, thereby limiting their ability to process large datasets. In this paper, we present SparkRA, an Apache Spark based pipeline to efficiently scale up the GATK RNA-seq variant calling pipeline on multiple cores in one node or in a large cluster. On a single node with 20 hyper-threaded cores, the original pipeline runs for more than 5 h to process a dataset of 32 GB. In contrast, SparkRA is able to reduce the overall computation time of the pipeline on the same single node by about 4×, reducing the computation time down to 1.3 h. On a cluster with 16 nodes (each with eight single-threaded cores), SparkRA is able to further reduce this computation time by 7.7× compared to a single node. Compared to other scalable state-of-the-art solutions, SparkRA is 1.2× faster while achieving the same accuracy of the results.

## 1. Introduction

With the development of next-generation sequencing (NGS) technologies, both DNA-seq and RNA-seq data are becoming increasingly accessible. Identifying variants from DNA-seq data attracted much attention from the research community, which resulted in the development of a number of tools and computational pipelines to address the problem. One of the most widely-used DNA-seq pipelines is GATK best practices [1], which recommends a sequence of tools to process DNA-seq data from raw reads all the way to variant calls. However, it usually takes many hours to run the complete pipeline, due to the large size of the input data and the poor scalability of some of the tools used.

In order to improve the performance of DNA pipelines and get results faster, a number of solutions have been proposed: Either by scaling the pipelines on multiple compute nodes in a cluster, or by improving the performance on a single node. In terms of cluster solutions, Churchill [2] adapts GATK and creates a high-performance alternative pipeline with an integrated set of tools to utilize the computational resources more efficiently. Other solutions, like Halvade’s [3], scale up the pipeline using the big data Hadoop MapReduce [4] framework. In contrast, SparkGA [5,6,7] is based on the in-memory big data framework Spark [8]. Both Halvade and SparkGA try to optimize the scalability of the GATK pipeline at a cluster level. The logic behind each of them is the same: The input data are divided into chunks and each step in the pipeline can be performed on those independent chunks to achieve data-parallel computations. The advantage of these two solutions is the ease of implementation of the pipelines on a scalable cluster without having to know the exact details of the cluster setup. In terms of performance improvement on a single node, various solutions have been proposed, ranging from algorithmic optimizations [9] to hardware acceleration [10].

At the same time, RNA-seq data have emerged as a cheaper and more efficient alternative to DNA sequencing data. While it has primarily been used for novel gene identification, expression quantification and splicing analysis [11], new analysis methods allow using this RNA-seq data for calling variants in the genome. In addition to the lower sequencing costs, variants discovered from RNA-seq data are from expressed genome regions, which provides direct evidence to study the relation between genotypes and phenotypes [12]. Furthermore, calling variants in RNA-seq is also an efficient option to validate the discoveries from whole-genome sequencing (WGS) or whole-exome sequencing (WES) experiments [13].

A number of tools and pipelines have specifically been proposed to enable RNA-seq data analysis; examples are GSNAP [14], MapSplice [15], TopHat [16] and STAR [17], which address the challenge of spliced reads alignment. In addition, the GATK team released their own best practices computational pipeline tailored for RNA-seq variant discovery [18]. However, variant calling in RNA-seq data has some unique computational challenges, such as the large difference in coverage between chromosomes, and the fact that the reads in RNA-seq data are not contiguous makes alignment and subsequent processing steps more difficult. This results in increasing the processing time of such data. For example, running the GATK RNA-seq pipeline on two paired-end data files of 25 GB each takes about 29 h on a single-core computer. On a system with 20 cores, the pipeline still takes up to 16 h, resulting in a speedup of about 2× rather than the desired 20×. This indicates the poor scalability of this pipeline, and the low efficiency of running it on multiple cores.

In order to address this issue of limited scalability, Halvade introduced an updated version of their pipeline called Halvade-RNA [19]. This solution starts by dividing the input data into chunks and then runs the GATK RNA-seq pipeline on each of these chunks independently to achieve data-parallel computations. A drawback of Halvade-RNA is that it uses the slow in-disk Hadoop MapReduce computation model, which introduces a large overhead on the computation time of the pipeline.

In this paper, we present SparkRA (Spark RNA Analysis), a pipeline that enables the GATK RNA-seq pipeline to scale efficiently on multiple nodes in a cluster environment. SparkRA uses the Apache Spark big data in-memory framework to facilitate easy scalability of the pipeline, while ensuring low computational overhead, thereby increasing the performance of the pipeline compared to other state-of-the-art solutions.

The contributions of this paper are:

1. Introducing SparkRA, a Spark-based pipeline to scale up RNA-seq analysis pipelines easily and efficiently, using the Apache Spark in-memory framework.
2. Improving the parallelism of the GATK best practices RNA-seq tools by addressing their sequential bottlenecks, and allowing them to take full advantage of the capabilities of SparkRA.
3. Comparing the performance of SparkRA with other state-of-the-art pipelines, and measuring an overall speedup of 7.7× as SparkRA scales the GATK RNA-seq pipeline from one node to 16. Compared to Halvade-RNA, our solution is about 1.3× faster on a single node and 1.2× faster on a cluster.

## 2. Methods

In this section, we discuss the details of SparkRA and how it is able to achieve efficient parallel scalability. First, we present the original GATK RNA-seq pipeline, then we discuss the parallelization and optimization techniques we implemented.

### 2.1. GATK RNA-seq

**Figure 1:**
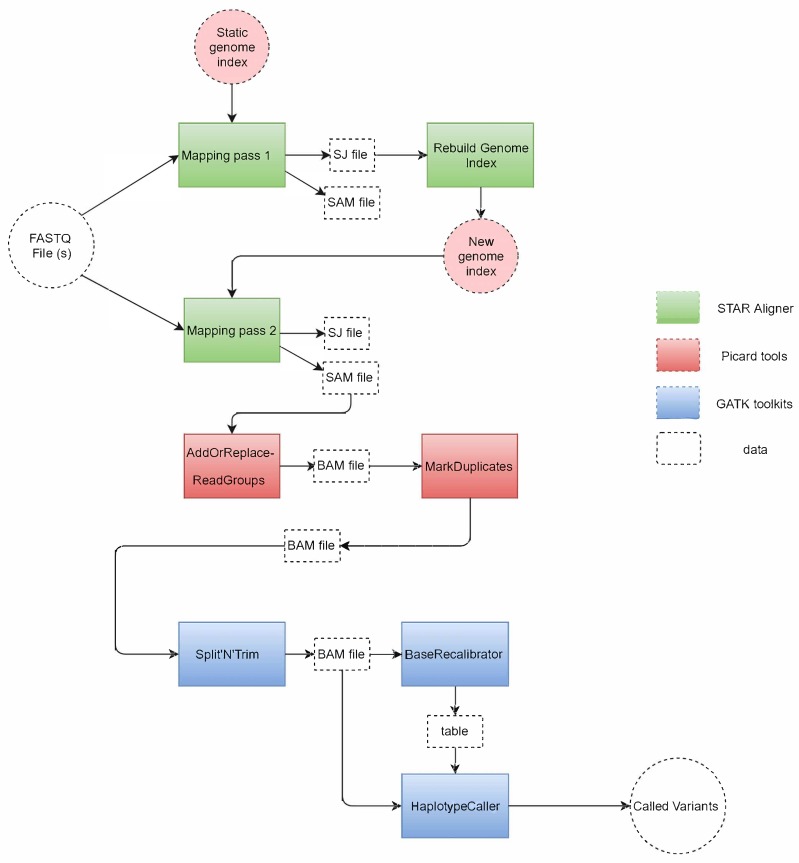
Tools used in the GATK RNA-seq pipeline and the way they process the data from input to output.

Figure 1 shows the tools used in the GATK RNA-seq pipeline and how they process the input files to variant calls at the output. The pipeline is divided into three main parts as highlighted in the figure, each using the following tools: 1. STAR aligner, 2. Picard tools, 3. GATK toolkit.

The pipeline starts by taking one (or two for paired-end reads) FASTQ file at the input. This file is read by the STAR aligner [17], which is considered as the most accurate aligner for RNA-seq reads [20]. STAR compares the reads to a reference genome encoded in a static genome index file to perform the first mapping pass (mapping pass 1). This mapping produces a SAM output file that contains the mapping location of each read to the reference. This file is discarded since this mapping is not accurate as it does not take into consideration that these RNA reads are spliced (i.e., split) at multiple intermediate locations (so-called junctions). In addition to the SAM file, a splice junction (SJ) file containing the splice junction information is produced by the first mapping pass. The SJ file is used by STAR as a guide in the rebuilding of the genome index (rebuild genome index), which produces a new genome index that includes the needed splice junction information to perform a more accurate mapping of the spliced reads in the FASTQ file. Then, STAR starts a second mapping pass (mapping pass 2) to create an accurate SAM file with read mapping information.

This SAM file is then used as input to Picard, the second tool in the GATK RNA-seq pipeline. First, Picard sorts the SAM file and creates groups of reads (AddOrReplace-ReadGroups) and compresses SAM to a BAM file for better performance in the rest of the pipeline. Then, Picard identifies repeated reads in the file and marks them as duplicates (MarkDuplicates) in the BAM file.

The remaining steps in the pipeline are carried out by various tools in the GATK toolkit. First, the Split’N’Trim tool is designed to identify the location of gaps in RNA reads, and to subsequently split a spliced read into exon segments. Then, the BaseRecalibrator tool creates a table to reassign the base quality values of the reads that could be biased by the sequencing machines. Finally, the pipeline ends by the HaplotypeCaller which is a variant calling tool that reads the BAM file and the base quality table to identify the probability of a variant in the reads with respect to the reference, and write these variants to a VCF file.

**Figure 2:**
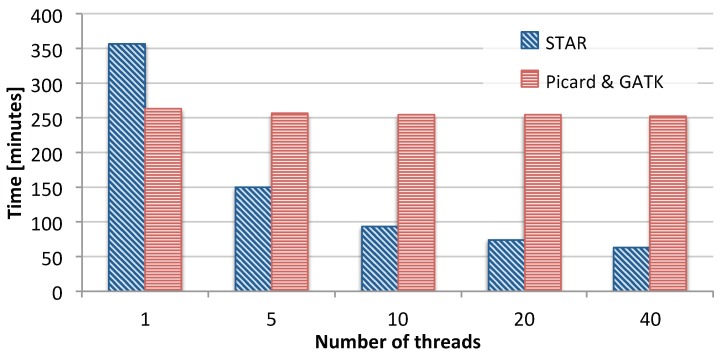
Scalability bottlenecks of the different tools in the GATK RNA-seq pipeline.

In order to identify the scalability bottlenecks in this pipeline, we measured the time taken by the different tools as we scale the number of available CPU threads the tools can use. Figure 2 shows the measurement results for running STAR on the one hand, and for Picard and GATK combined on the other. Throughout the paper, Picard and GATK are treated as a single component in the pipeline, which a feature of our scalability framework. The figure shows that as the number of threads increases, STAR is able to effectively make good use of these threads and reduce its runtime from about 350 min down to about 70 min. However, the runtime of Picard and GATK hardly ever changes as we increase the number of threads. This is due to the fact that among all the tools used in Picard and GATK, only the BaseRecalibrator can be executed with more than one thread.

**Figure 3:**
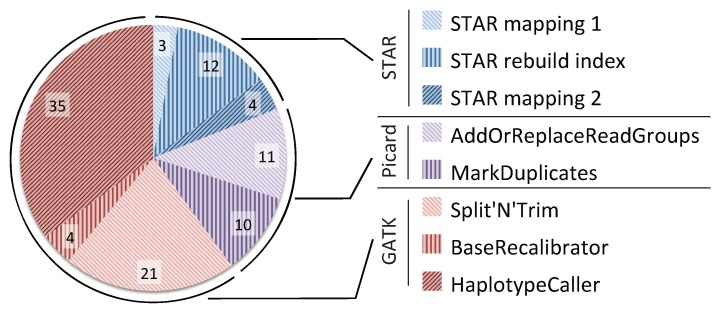
Percentage of time taken by each tool in the GATK RNA-seq pipeline on a single node with 40 threads.

In addition to evaluating the scalability potential of the pipeline, we also investigated the computational bottlenecks of the pipeline. Figure 3 shows the percentage breakdown of the time taken by each tool in the GATK RNA-seq pipeline when executed on a single node with 40 threads. The figure shows that STAR is the fastest part of the pipeline, taking about 19% of the total time, 12% of which is used for index rebuilding. Picard is slower, taking 21% of the total time. Finally, the GATK toolkit takes most of the runtime consuming 60% of the pipeline. The results in Figure 2 and Figure 3 show that in order to get any benefit from scaling up our pipeline to multiple nodes, we will have to address the limited scalability of Picard and GATK.

### 2.2. SparkRA Execution Flow

**Figure 4:**
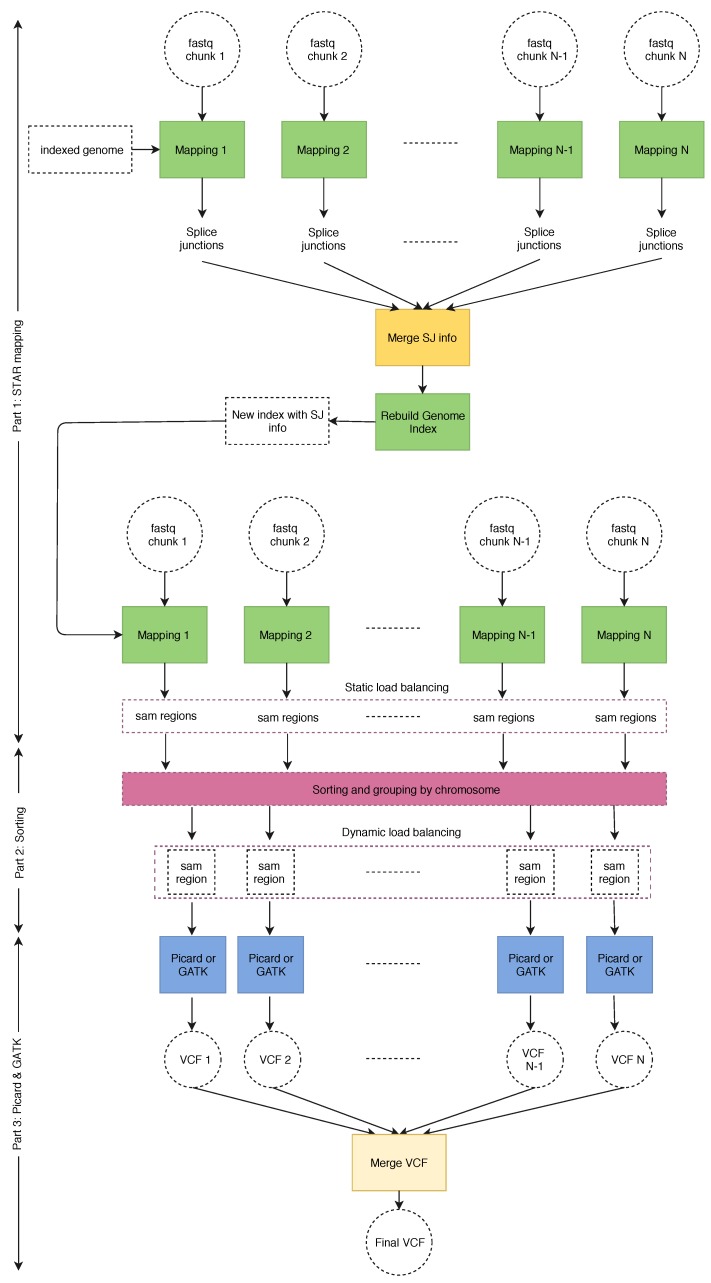
Execution flow of the SparkRA pipeline.

Figure 4 shows the general execution flow of our SparkRA pipeline [21]. The general idea behind parallelizing the various tools is to divide the input files of each tool into a number of chunks, and then running multiple instances of each tool in parallel to allow for efficient scalability to multiple threads and multiple nodes. However, there are a couple of challenges that limit the parallel potential of the pipeline. First of all, there are three points in the pipeline (1. merge SJ info, 2. sorting and 3. merge VCF) where the next tool needs to wait on all the instances of the previous tool to finish completely before it can start. These points are called synchronization points. In order to reduce their impact, we need to balance the execution time of the tool instances running in parallel so that they finish in approximately the same time, such that waiting time can be minimized. Secondly, the sorting task itself has a strong sequential component and is therefore difficult to parallelize. As a result, we need to implement it in a very efficient way to ensure that it does not become a bottleneck for the pipeline as it scales.

Figure 4 shows the various components of the SparkRA pipeline. Before starting the pipeline, the FASTQ files are divided into chunks of nearly equal size. The initial version of the genome index is stored locally on all nodes to allow all tasks to access it. Then multiple instances of STAR aligner are executed to map the reads in the chunks to the reference in parallel. The number of chunks is equal to the number of threads available on the cluster. Next, the first synchronization point is reached, where the splice junction files must be merged into one and used to rebuild the genome index. STAR then uses the new genome index to remap the FASTQ chunks to the reference. The output SAM strings of the second mapping pass have to be sorted and grouped by chromosome before being fed to Picard and GATK. This is the second synchronization point. After grouping by chromosome, bigger chromosomes are divided into regions to even out the distribution of the reads in the SAM file. This creates smaller SAM files with chromosome regions to ensure better load balancing of later stages of the pipeline. Finally, each SAM region is processed by Picard and GATK to identify variant calls, stored in multiple VCF files. This is where the third synchronization point is reached. These VCF files are then merged into one final VCF file used for further analysis.

The SparkRA pipeline has been implemented in Spark, using the Scala language, with minor utilities written in Java and Python. To maximally use the parallelism available in the pipeline, we implement two load balancing techniques (static and dynamic load balancing) to allow the different parallel processes to overlap their execution as much as possible. The pipeline is separated into three parts.

Part 1 does the alignment and performs static load balancing according to the size of each chromosome defined by the reference genome.
Part 2 performs dynamic load balancing to divide the SAM files according to the actual number of reads mapped to each chromosome.
Part 3 is represented by Picard and GATK.

In Part 1, there are two optimizations we can perform to run STAR efficiently. First, since we execute multiple STAR instances, each instance requires to load the full genome index file into memory (about 27 GB of RAM for human RNA), which occupies excessive amounts of memory. In order to reduce memory utilization, STAR allows loading the genome index to a shared memory on each node that can be used by all STAR instances on that specific node. Second, since rebuilding the genome index is a totally sequential step, it becomes an increasingly serious bottleneck as the pipeline scales. Therefore, STAR allows to rebuild an index with a sparse suffix array in a sparse way, which can be built much faster. For example, a suffix array of sparsity 8 (i.e., distance between indices is 8) is 6.3× faster to build than a suffix array of sparsity 1 (39.2 min versus 6.27 min). A sparse suffix array does, however, slow down the second mapping, but since the mapping can be parallelized, it is not a bottleneck for scalability.

### 2.3. Static and Dynamic Load Balancing

As shown in Figure 4, Part 3 (Picard and GATK) of the pipeline is executed fully in parallel. This means that it is important to balance the load of each of the parallel instances to minimize the overall computation time. This can be done by balancing the number of reads in every SAM region provided as input to each pipeline instance in Part 3. Since this in itself a computationally intensive process to be done for the many millions of reads in the SAM file, we divide the process into two stages: Static and dynamic load balancing, as presented by SparkGA [5].

#### 2.3.1. Static Load Balancing

If we assume that the reads are on average distributed equally across the different chromosomes in the DNA, we can approximate the load balancing process by dividing the reads in the SAM file according to the size of the DNA region they belong to. Since this information is known in advance (before the pipeline starts), we can perform it statically for a specific reference genome. If ${\mathrm{sizeStatic}}_{\mathrm{sum}}$ represents the total size of the genome in base pairs, and numRegions represents the number of regions we would like to create (based on the number of parallel instances of the pipeline we would like to create), then the target size of each region used for static load balanced would be

(1) $$\mathrm{avgSizeStatic}=\frac{{\mathrm{sizeStatic}}_{\mathrm{sum}}}{\mathrm{numRegions}}$$

However, to make the computation easier, we first create regions along the boundaries of individual chromosomes. Then, we only divide a single chromosome into two or more regions if the size of the chromosome is larger than a multiple of numRegions. This means that the eventual number of regions actually created is not equal to the targeted numRegions. We refer to the number of regions actually created during static load balancing as $R_{sLB}$.

Static load balancing requires very limited computation, since all the needed information is already available after STAR mapping of Part 1 completes. This means that we can use this simple heuristic already in Part 1 to improve the performance of Part 3 at a negligible cost. Moreover, the performance of Part 3 can be further improved using dynamic load balancing in Part 2 as discussed in the next section.

#### 2.3.2. Dynamic Load Balancing

Static load balancing is performed without taking in account the actual number of reads mapped to every region of the genome. This is done to simplify the computational complexity of the balancing process. However, after the alignment and sorting takes place, we perform a second load balancing iteration based on the actual number of mapped reads in every region to further optimize the process. This second iteration is referred to as dynamic load balancing, since it uses the mapping information that was just calculated in Part 1 of the pipeline.

The actual total number of reads (${\mathrm{sizeDynamic}}_{\mathrm{sum}}$) can efficiently be calculated in Spark at run time. The target size of each genome region (avgSizeDynamic) can be calculated as follows.

(2) $$\mathrm{avgSizeDynamic}=\frac{{\mathrm{sizeDynamic}}_{\mathrm{sum}}}{R_{\mathrm{sLB}}}$$

Then, this number is compared with the actual number of reads in each genome region. If the number of reads is larger than a multiple of avgSizeDynamic, we will divide the genome region into sub-regions to make the load more balanced for Part 3 of the pipeline. We refer to the number of regions actually created during dynamic load balancing as $R_{dLB}$.

In many cases, the number of reads in the majority of regions will not exceed a multiple of avgSizeDynamic. Therefore, this step will not incur much delay in the pipeline. However, in cases where the number of reads in a region does happen to be rather high, dynamic load balancing will succeed in preventing this region from becoming a bottleneck for Part 3 of the pipeline.

## 3. Results

In this section, we discuss the measurement results of our SparkRA pipeline as compared to other state-of-the-art solutions. In these experiments, we use a dataset with two files of 16 GB each: ENCFF005NLJ and ENCFF635CQM. The data are publicly available from the Encyclopedia of DNA Elements (ENCODE) [22]. We used GATK version 3.4 instead of GATK version 4, since the cluster used to run the experiments did not support running GATK version 4.

First, we present the scaling up results on a single compute node, followed by the results on multiple nodes in a cluster.

### 3.1. Single-Node Performance

For the single node experiment, we run the pipelines on a node that has two processors, each with 10 physical cores with hyper-threading enabled (two threads per core). The node is able to run 40 threads and has 196 GB of RAM.

#### 3.1.1. Impact of Load Balancing

Table 1 lists the relationship between the number of regions requested by the user and the actual number of generated regions during static and dynamic load balancing. The results show that there are always more static regions generated than the requested number of regions. This takes place due to the large difference between the sizes of chromosomes in the human genome. Since we start by creating regions based on chromosomes and subsequently divide these into sub-regions, smaller chromosomes will always have their own region and more regions are added by dividing bigger chromosomes into sub-regions. This effect diminishes gradually as we increase the number of requested regions. The table also shows that there are even more dynamic regions created than static ones (36% to 56% more). This indicates that the number of reads is not uniformly distributed along the genome, but comes rather concentrated in specific regions. This is specifically true for RNA-seq data, as it gets expressed in specific genetic regions only.

With respect to computation time, the shortest total time is achieved with 72 requested regions. However, the table shows limited impact of the change in the number of regions on the total time. There is a slight decrease in the time of Part 2 as the number of regions increases, which gets offset by a slight increase in the time of Part 3. The limited impact of the number of regions here can be attributed to the limited amount of parallel resources a single node has. Parallelization can help up to the limit of the nodes capabilities. Since our node has 20 cores capable running a total of 40 threads, creating up to 40 regions can help reduce the computation time of the pipeline. However, with 50 regions or more, the computation time is not expected to decrease much. The increased number of regions beyond 40 is chosen here to investigate the impact on the actual number of regions generated during static ($R_{\mathrm{sLB}}$) and dynamic ($R_{\mathrm{dLB}}$) load balancing.

**Figure 5:**
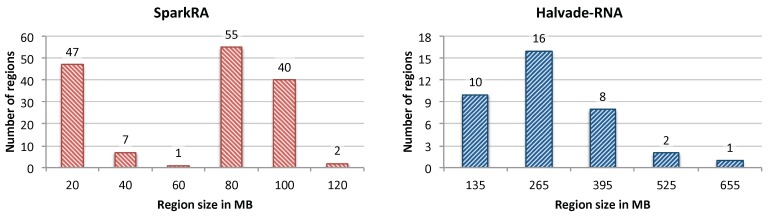
Histogram of the size of regions (in MB) generated by SparkRA (**left**) and Halvade-RNA (**right**).

To show the effectiveness of our load balancing technique, we compare it with the load balancing method used by Halvade-RNA. Figure 5 shows a histogram of the size of regions (in MB) generated by SparkRA (left) and Halvade-RNA (right). The static and dynamic load balancing technique used by SparkRA insures that enough regions are generated (depending on the size of the input data), such that the maximum sizes of the regions remain relatively small. This is not the case for Halvade-RNA, which uses a fixed number of regions irrespective of the size of the data. The figure shows that the maximum size of the regions generated by SparkRA for the used dataset is less than 120 MB, while regions can have a size of up to 655 MB for Halvade-RNA. This difference is made possible due to two reasons. On the one hand, SparkRA is able to generate many more regions (161 in this case) compared to Halvade-RNA (only 37 regions) when the size of the dataset is large, thereby keeping the size of each region smaller. On the other hand, SparkRA specifically breaks down larger regions into smaller ones, rather than uniformly dividing the whole DNA in equal sized regions. This results in creating more regions with larger sizes, which increases the utilization of the compute infrastructure, and reduces the number of CPUs that wait idle after processing the smaller regions. The figure shows that most of the regions generated by Halvade-RNA have sizes smaller than 50% of the corresponding maximum region size, while most of the regions generated by SparkRA have sizes larger than 50% of the corresponding maximum region size.

#### 3.1.2. Comparison with Existing Solutions

In this section, we compare the compute capabilities of SparkRA with the computation time of other state-of-the-art solutions in the field: The original GATK RNA-seq pipeline and Halvade-RNA, as shown in Table 2. The table lists two measurements for GATK: Executed with only 1 thread (GATK1) and with 40 threads (GATK40). The table also lists the time taken by the different parts of each pipeline. For Part 1 (STAR mapping), we provide the time for the first and second STAR aligner passes, in addition to the genome index rebuild step in between. For Part 2 (sorting and dynamic load balancing), we provide only a measurement for SparkRA; the sorting time for GATK and Halvade is included in the time of Part 3 in the table.

**Figure 6:**
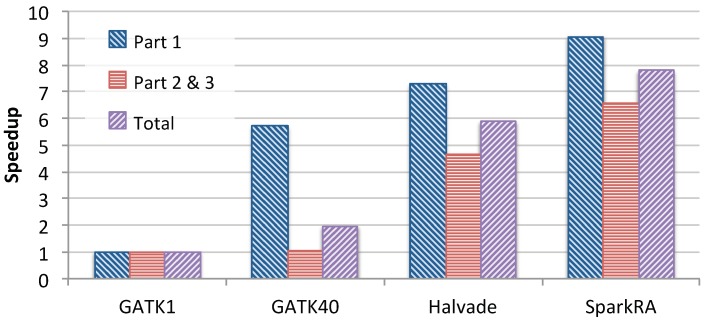
Single-node speedup of 40-thread GATK RNA-seq (GATK40), Halvade-RNA and SparkRA over single-threaded GATK RNA-seq (GATK1).

Figure 6 shows the speedup achieved by the 40-thread GATK RNA-seq pipeline, Halvade-RNA and SparkRA over the single-threaded GATK pipeline. We compare Parts 1 and 3 of the pipeline in addition to the total speedup. For SparkRA, we compare the combined time of Parts 2 and 3 to that of Part 3 of GATK1. The figure shows that SparkRA is by far the fastest pipeline, achieving a speedup of 7.8×, followed by Halvade with 5.9×, and finally GATK40 with about 2×, compared to GATK1. This same is true for Parts 1 and 3 of the pipelines individually. Compared to Halvade, SparkRA is about 1.32× faster in total, 1.4× faster for Parts 2 and 3 and 1.24× faster for Part 1. These results indicate that SparkRA makes good use of the parallel capabilities of Spark and allows for easy scalability on a single node.

Interestingly, Table 2 shows that SparkRA is actually slower than GATK40 for both STAR pass 1 and 2. This has two reasons. On the one hand, STAR is implemented to scale very efficiently on a single node with increasing number of threads (as shown in Figure 2). On the other hand, Spark scalability is done by creating multiple independent STAR processes, which creates much more overhead, in addition to extra overhead that the Spark framework itself adds to the computation time to manage the scheduling and resilience of these processes.

### 3.2. Multi-Node Performance

This section discusses the scalability capabilities of SparkRA in a cluster environment. We use SURFsara, the Dutch national supercomputing infrastructure to run our analysis [23]. Each node we used on the SURFsara cluster has 56 GB of RAM and two processors, each with four single-threaded cores (total eight cores per node). The cluster uses the Hadoop Distributed File System (HDFS) for distributed file storage. We tested SparkRA on 1, 4, 8 and 16 nodes.

#### 3.2.1. Scalability of SparkRA

**Figure 7:**
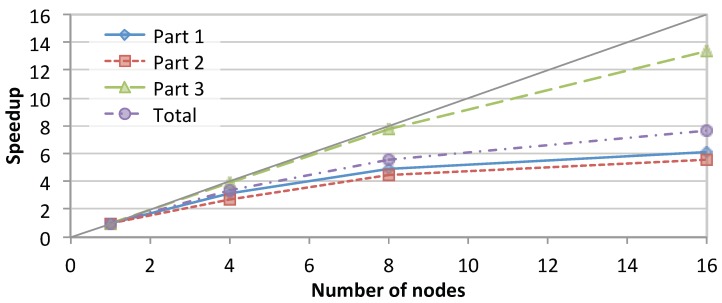
Speedup achieved by SparkRA and its parts when running on 1, 4, 8 and 16 nodes in the cluster.

Figure 7 shows the total speedup achieved by SparkRA and each of its parts on the cluster while running on 1, 4, 8 and 16 nodes. The figure shows that the total speedup of SparkRA scales up to 7.7× for 16 nodes, with Part 3 achieving the highest speedup of 13×, followed by Part 1 with 6.1× and Part 2 scaling the slowest with 5.6×. The high scalability of Part 3 can be attributed to the large amount of parallelism available in the algorithm. The only sequential component in Part 3 is at the end of the pipeline where the relatively small VCF files need to be combined. Part 1 also has a lot of parallelism, but also has a significant sequential component represented by rebuilding the genome index, which reduces the overall scalability of Part 1. The least scalable part of the pipeline is Part 2, which has a communication bottleneck, as the sorting step requires the location of mapped reads computed on a given node to be communicated and compared with the mapped location of all other reads in other nodes on the cluster. This significantly limits the scalability potential, since increasing the number of nodes also increases the number of sources and destinations to communicate with. However, since the time taken by Part 2 is relatively small, it has limited impact on the overall speedup. Still, if we increase the number of nodes even further beyond 16 nodes, this part will eventually become an important bottleneck.

#### 3.2.2. Comparison with Existing Solutions

Table 3 lists the time taken by Halvade-RNA and SparkRA on the SURFsara cluster, as well as the speedup achieved by SparkRA for the different parts of the pipeline. The table shows that SparkRA is 1.22× faster than Halvade for the total runtime of the pipelines, bringing the total runtime down from 80.9 to 66.5 min. For Parts 2 and 3, SparkRA is 1.36× faster than Halvade. This underscores the effectiveness of the load balancing capabilities of our pipeline, enabling it to benefit as much as possible from the parallelism in Part 3. For Part 1, our pipeline has a more modest speedup of 1.16× as a result of sequential limitations to scalability of that part.

## 4. Discussion

This section provides a discussion of the results and measurements and analyzes the bottlenecks and limitations of the SparkRA pipeline. We start by an analysis of the CPU utilization in the cluster mode, followed by a discussion of the accuracy of the pipeline results.

### 4.1. CPU Utilization

This section discusses in more details CPU utilization while SparkRA is running in cluster mode. This allows us to understand the efficiency of our solution and potential areas to improve performance even further.

**Figure 8:**
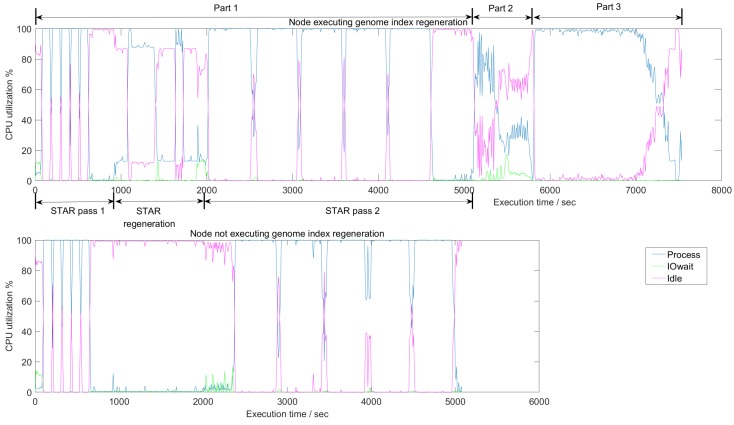
Average CPU utilization of the three different parts of SparkRA on a 16-node cluster. In Part 1, there are three steps: STAR pass 1, STAR index regeneration, and STAR pass 2. The top and bottom charts show utilization in a node with and without genome index regeneration, respectively. Parts 2 and 3 are not shown in the bottom chart, since they are identical to the top chart. Measurements are generated using the iostat profiler [24].

Figure 8 shows the average CPU utilization in a node on the cluster as it executes the different parts of SparkRA. The figure has three curves, one representing an active CPU (Process), a CPU waiting for I/O access (IOwait), and an idle CPU (Idle). There are two charts in the figure that show the utilization during the three parts of SparkRA. In Part 1, however, there are three steps: STAR mapping pass 1 done in parallel on all nodes, followed by an index regeneration step done sequentially on only one node in the cluster, and finally a second STAR mapping pass done on all nodes. The top chart in the figure shows utilization on the one node in the cluster doing all these three steps including the sequential index regeneration, while the bottom chart shows utilization of one of the other nodes in the cluster doing only the two parallel STAR mapping steps. Parts 2 and 3 take place on all nodes of the cluster, but are not shown in the bottom chart, since they are identical to the top chart.

Generally, the figure indicates that CPUs in the cluster are highly utilized throughout the execution of the pipeline, since “Process” is close to 100% utilization all the time. There are a number of exceptions, however, specifically during the index regeneration step in Part 1, and during Part 2.

In Part 1, we can clearly observe different utilization behaviors between mapping pass 1, the index regeneration and mapping pass 2. At the end of STAR pass 1, computation stops and splice junction information is sorted, shuffled and finally collected on one node. This explains the 100% “Idle” time at the end of the STAR pass 1. Then, during the index regeneration step, there is a period (1000 to 2000 s) where only one node has some “process” activity (top chart), while all other nodes are idle (bottom chart). This step ends with I/O wait activity (top chart) as the newly generated index is being copied to other nodes and gets stored in disk. Finally, the STAR pass 2 starts with I/O wait activity in all other nodes (bottom chart), as these nodes read the new index to use it in pass 2. Then there is a period of active STAR mapping, which ends Part 1 at about 5000 s.

In Part 2, sorting takes place which, as we discussed earlier, has a significant communication component. Therefore, the CPU utilization in this part is the least compared to the other pipeline parts. As the figure shows, the “Idle” curve is elevated throughout this part. In addition, the figure shows a consistently elevated “IOwait” curve, indicating a large I/O access and network access component in this part.

In Part 3, Picard and GATK tools are used to call variants in the reads. This figure shows that this part has the highest utilization as the “Process” curve is consistently at 100%. This result validates our earlier observation that Part 3 is the most parallelizable and scalable part in the pipeline. This high 100% utilization continues until 7000 s, which is where the VCF files are generated. Then utilization drops gradually until the files are combined together into one file at the end of the pipeline.

Based on these observations, future work can focus on Part 2 to improve CPU utilization during the sorting part. One possible solution is to partly overlap sorting with mapping in Part 1, by starting the sorting as the reads are mapped by the STAR aligner. Another possibility to improve performance is by optimizing the STAR index regeneration step in Part 1. However, this step is STAR specific and will require changes to the aligners itself.

### 4.2. Pipeline Accuracy

An important aspect of scaling up a genomics pipeline is to analyze the accuracy characteristics of the computed results. Due to dividing various intermediate files into chunks and the distribution of the computation on multiple nodes, some of the computations might be carried out differently in a distributed pipeline as compared to computing using a centralized pipeline. This happens primarily at the boundaries of genomic regions, since short reads that are commonly 150 base pairs long get sorted into different file chucks depending on their starting map position, without taking overlapping reads in two regions into consideration.

In this section, the accuracy is evaluated by comparing the variant calls in the VCF files calculated by a given pipeline, as compared to the VCF variants calculated by the baseline GATK pipeline. We define pipeline accuracy using two metrics: Sensitivity and precision. In order to calculate these two metrics, we need to measure the following parameters from the two VCF files: True positive (TP) variants, false positive (FP) variants and false negative (FN) variants. TPs represents variants called by both pipelines, FPs represent variants called by the scalable pipeline but not by GATK. FNs represent variants called by GATK but not by the scalable pipeline. Using these, we can calculate sensitivity and precision as follows:

(3) $$\mathrm{Sensitivity}=\frac{\mathrm{TP}}{\mathrm{TP}+\mathrm{FN}}$$

(4) $$\mathrm{Precision}=\frac{\mathrm{TP}}{\mathrm{TP}+\mathrm{FP}}$$

Table 4 lists the accuracy of VCF files generated by the cluster deployment of SparkRA and Halvade-RNA as compared with the GATK baseline in terms of precision and sensitivity. The table shows that in general both SparkRA and Halvade have similar sensitivity and precision of about 95% and 94%, respectively. At close inspection, we find that Halvade has a slightly higher sensitivity of 0.23 percentage points than SparkRA, and a slightly higher precision of 0.04 percentage points. This difference can be attributed to the significantly increased number of regions (29% more) created by SparkRA (183 regions) compared to Halvade (142 regions). The increased number of regions of SparkRA allows for the large amount of performance increase as compared to Halvade, albeit at a minor degradation in accuracy.

## 5. Conclusions

This paper introduced SparkRA, an RNA-seq analysis pipeline that allows for easy and efficient scalability of the GATK RNA-seq best practices pipeline using the Apache Spark big data framework. SparkRA scales up the computations by dividing data files into chunks that get processed in parallel by executing multiple instances of the GATK pipeline tools. It can help scale performance of the pipeline both on a single compute node, and on a large compute cluster. Our solution is able to limit the imbalance in the computation time between different chunks of the data using a fast heuristic consisting of two load balancing stages: Static and dynamic load balancing. This approach allows for load balancing that is both fast as well as fine tuned to the input data. Experimental results show that SparkRA is effective in utilizing CPU resources keeping CPU of all nodes active at close to 100% utilization most of the time. SparkRA can achieve 7.8× speedup on a single node compared with a single thread run of the GATK pipeline. On a cluster of 16 nodes, SparkRA can achieve further speedup of 7.7× compared to a single node. Compared to existing state-of-the-art solutions, SparkRA is about 1.2× faster, while achieving the same accuracy of results.

## Figures and Tables

**Table 1:** Impact of the user requested number of the regions (numRegions) on the actual number of regions generated during static ($R_{\mathrm{sLB}}$) and dynamic ($R_{\mathrm{dLB}}$) load balancing, in addition to the resulting total computation time of the pipeline and that of Parts 1 to 3 (single-node performance).

			Time [minutes]			
numRegions	$R_{\mathrm{sLB}}$	$R_{\mathrm{dLB}}$	Part 1	Part 2	Part 3	Total
180	224	304	39.4	9.33	32.5	81.2
144	193	262	39.7	10.3	30.8	80.8
108	156	217	40.7	11.2	29.2	81.0
72	123	183	39.7	11.4	29.1	80.2
50	103	161	40.0	13.8	29.4	83.2

**Table 2:** Pipeline computation time (in minutes) comparison of GATK RNA-seq running with one thread (GATK1) and 40 threads (GATK40) with Halvade-RNA and SparkRA (single-node performance).

Pipeline Stage		GATK1	GATK40	Halvade	SparkRA
Part 1:	STAR (pass 1)	92.4	9.87	9.60	10.5
	Index rebuild	152	39.2	6.70	6.27
	STAR (pass 2)	116	14.0	33.0	22.9
	Total Part 1	360	63.1	49.3	39.7
Part 2:	Sorting	n/a	n/a	n/a	11.4
Part 3:	Picard & GATK	265	255	56.8	29.1
Total pipeline time		625	318	106	80.2

**Table 3:** Pipeline computation time (in minutes) results and speedup of SparkRA compared to Halvade-RNA running on the SURFsara cluster.

Pipeline Stage	Halvade	SparkRA	Speedup
Part 1: Mapping	54.7	47.2	1.16
Parts 2 and 3: Sorting, Picard & GATK	26.2	19.3	1.36
Total pipeline time	80.9	66.5	1.22

**Table 4:** Accuracy of VCF files generated by the cluster deployment of SparkRA and Halvade-RNA as compared with the GATK baseline in terms of sensitivity and precision. The comparison results were generated using RTG Tools [25].

Pipeline	#Regions	TP	FP	FN	Sensitivity	Precision
SparkRA	183	109411	6886	6363	94.50%	94.08%
Halvade	142	109669	6850	6105	94.73%	94.12%
